# Does the Vaginal Microbiota Influence the Incidence of the Preterm Premature Rupture of Membranes?

**DOI:** 10.3390/jcm14186577

**Published:** 2025-09-18

**Authors:** Stepan Feduniw, Natalia Zeber-Lubecka, Michal Pruc, Zuzanna Gaca, Łukasz Szarpak, Michal Ciebiera

**Affiliations:** 1 Obstetrics & Gynecology Department, Spital Uster, 8610 Uster, Switzerland; 2 Department of Gastroenterology, Hepatology and Clinical Oncology, Centre of Postgraduate Medical Education, Roentgena 5, 02-781 Warsaw, Poland; natalia.zeber-lubecka@cmkp.edu.pl; 3 Department of Genetics, Maria Sklodowska-Curie National Research Institute of Oncology, 00-001 Warsaw, Poland; 4 LUXMED Group, Department of Clinical Research and Development, 02-676 Warsaw, Poland; m.pruc@ptmk.org (M.P.); lukasz.szarpak@gmail.com (Ł.S.); 5 Research Unit, Polish Society of Disaster Medicine, 05-806 Warsaw, Poland; zuzanna.k.gaca@gmail.com; 6 Department of Public Health, International European University, 02091 Kyiv, Ukraine; 7 Henry JN Taub Department of Emergency Medicine, Baylor College of Medicine, Houston, TX 77030, USA; 8 Second Department of Obstetrics and Gynecology, Centre of Postgraduate Medical Education, Inflancka 6, 00-189 Warsaw, Poland; michal.ciebiera@gmail.com; 9 Warsaw Institute of Women’s Health, 00-189 Warsaw, Poland

**Keywords:** preterm premature rupture of membranes, PPROM, microbiome, vaginal flora

## Abstract

**Introduction**: The study aimed to provide a systematic review and analysis of previously reported studies investigating the association between the bacterial microbiome and the incidence of preterm premature rupture of membranes (PPROM). **Material and Methods**: A comprehensive literature search across many databases via 01 March 2023, including PubMed, Web of Science, Embase, and the Cochrane Library. **Results**: A total of 20 studies were reviewed, all of which provided a comprehensive analysis of the microbial makeup in pregnant women. The findings suggest that disturbances in the bacterial microflora correlate with a heightened risk of PPROM. **Conclusions**: There was a significant reduction of naturally prevalent vaginal species (in the vaginal flora of women with PPROM such as *Lactobacillus* spp., *Weissella* spp., and *Rickettsiales* spp. This was accompanied by the dominance of other bacterial species such as *Sneathia* spp., *Prevotella* spp., *Prevotella bivia*, *Prevotella timonensis*, *Peptniphilus*, *Streptococcus* spp., *Dialister* spp., *Lactobacillus iners*, *Gardnerella vaginalis*, *Ochrobactrum* spp. *Megasphaera* spp., *Faecalibacterium* spp., *Bifidobacterium* spp., *Xanthomonadales* spp., *Gammaproteobacteria* spp., *Alphaproteobacteria* spp., *Bacteroides* spp., *Sphingomonas* spp., *Streptococcus agalactiae*, *Escherichia coli*, *Staphylococcus aureus*, *Chlamydia trachomatis*, *Ureaplasma urealyticum*, *Ureaplasma parvum* or Group B *Streptococcus* begin to dominate, leading to PPROM. Recognising the microbial patterns could lead to the development of risk-based microbiological interventions and probiotic treatment, potentially improving the management and outcomes of patients with PPROM.

## 1. Introduction

The vaginal microbiome plays an essential role in pregnancy. Throughout pregnancy, hormonal variations, especially changes in estrogens and progesterone, can influence the composition of the vaginal microbiota, potentially influencing maternal and fetal health [1]. The term “microbiome” refers to the community of microorganisms, including bacteria, fungi, and viruses, present in the vagina. A healthy vaginal environment is usually dominated by *Lactobacillus* species, which helps maintain a slightly acidic pH and prevents the overgrowth of pathogenic bacteria [2].

Hormonal fluctuations during pregnancy can alter the vaginal microbiome, leading to a reduction in the levels of *Lactobacillus* species. This, in turn, can result in a less varied and stable microbiome [3]. It is also important to note that the composition of the vaginal microbiome varies throughout a woman’s life—it is different in preadolescence, in reproductive age and in postmenopausal women [4]. This shift in the composition of the vaginal microbiome might increase the risk of certain conditions, such as bacterial vaginosis (BV) and urinary tract infections [5].

Maintaining a balanced vaginal microbiome is considered crucial for a healthy pregnancy [6]. Vaginal dysbiosis, either increased microbial diversity or the presence of pathogenic bacteria, has been linked to an increased risk of preterm birth (PTB), preterm premature rupture of membranes (PPROM), and other complications like intrauterine infection or chorioamnionitis [7].

PPROM refers to the rupture of the amniotic sac before 37 weeks of gestation (WG) [8]. Emerging evidence suggests the microbiome, including the vaginal microbiome, might play a role in PPROM [9]. The amniotic fluid, which surrounds and protects the developing fetus, was traditionally thought to be sterile. However, recent studies have shown it can contain microorganisms, especially in women with PPROM [10]. Interestingly, these microbes are not observed in the amniotic fluid of women with healthy term pregnancies [11]. Microbes can reach the amniotic cavity through various routes, including infections ascending from the lower genital tract [10]. Bacterial Vaginosis (BV) is characterized by a reduction in beneficial *Lactobacillus* species coupled with an overgrowth of pathogenic bacterial populations. The presence of certain bacteria associated with BV, including *Gardnerella vaginalis* and *Mycoplasma hominis*, has been linked to an elevated risk of PPROM [12]. Vaginal dysbiosis and the presence of specific pathogenic bacteria can trigger an inflammatory response in the reproductive tract, weakening the fetal membranes, thus increasing the risk of PPROM. Local inflammation in the region of the fetal membranes leads to elevated levels of pro-inflammatory cytokines and matrix metalloproteinases, which weaken the membrane by degrading its structural components. In addition, dysbiosis-induced oxidative stress promotes the accumulation of reactive oxygen species, further compromising the integrity of the fetal membranes by directly damaging collagen and extracellular matrix proteins.

Although there is currently limited research on the direct relationship between the endometrial and intestinal microbiome and PPROM, the concept of an “endometrial microbiome” is a topic of active research, and our understanding is evolving. The uterus has been perceived to be a sterile environment. However, recent findings suggest the presence of a low abundance microbial community in the endometrium [13]. Research on the relationship between PPROM and the endometrial microbiome remains limited, and the specific relationship is not yet well-established. Most studies have primarily focused on the vaginal microbiome rather than the endometrial microbiome in relation to PPROM. However, some studies suggest a potential connection between an altered endometrial microbiome and adverse pregnancy outcomes, including preterm birth [14]. It is believed that disrupting the physiological microbial balance in the endometrium might contribute to inflammation and other pathological processes, increasing the risk of PPROM. Furthermore, alterations in the intestinal microbiome could result in inflammation or immune dysregulation, potentially influencing pregnancy outcomes, including PPROM [15]. As research in this area is still in its early stages, it is important to recognize the need for further studies to understand better the relationship between the endometrial and intestinal microbiome and PPROM. Therefore, clinical management of PPROM primarily focuses on diagnosing and managing the condition rather than directly targeting the endometrial microbiome.

The study aimed to provide a systematic review and analysis of previously reported studies investigating the association between the bacterial microbiome and the incidence of preterm premature rupture of membranes (PPROM).

## 2. Materials and Methods

### 2.1. Study Design

The present study followed the Preferred Reporting Items for Systematic Reviews and Meta-Analyses (PRISMA) guidelines, adherence to these guidelines is further documented in Supplementary Table S1 [16]. Prior to commencing the investigation, the research protocol underwent approval by all authors and was subsequently registered in the PROSPERO registry (International Prospective Registry of Systematic Reviews) under the assigned registration number CRD42023463362.

### 2.2. Search Strategy

Articles deemed potentially eligible were independently assessed by two reviewers (M.P. and S.F.). In case of discrepancy, further discussion was conducted, and the third reviewer was consulted (N.Z.-L.). Comprehensive research was conducted across many databases via 1 March 2023, encompassing PubMed, Web of Science, Embase, and the Cochrane Library. Additionally, Google Scholar was searched and used as a supplementary electronic repository. The researchers used a combination of the following keywords: “microbiota” OR “microbiome” OR “microfilm” OR “microflora” OR “microorganism” OR “High-Throughput Nucleotide Sequencing” OR NGS OR “next-generation sequencing” OR “metagenomic” OR “16S RNA” AND “premature rupture of membranes” OR “preterm premature rupture of membranes” OR “preterm prelabour rupture of membranes” OR PROM OR PPROM. The reference lists of the studies that were included in the analysis were carefully examined to identify potentially relevant publications. In order to avoid redundancy, only the most recent or comprehensive reports authored by the same individuals were included in the analysis. In addition, the reference lists of included articles and systematic reviews were examined for potentially relevant papers. The references were imported into Mendeley (version 1.19.8), and any duplicate entries were subsequently eliminated.

### 2.3. Inclusion and Exclusion Criteria

Our analysis included studies that investigated the association between various microbial species in the vaginal microflora and the incidence of PPROM. Eligible studies were required to meet the following inclusion criteria: a study comparing different microbiological spaces in pregnant women with PPROM to a control without PPROM. Studies that matched the following criteria for exclusion were not included: (1) studies not yielding the specified outcome; (2) studies without a comparable cohort; (3) studies not published in English; (4) articles where PPROM was not confirmed or not appropriately diagnosed; and (5) editorials, conference papers, reviews, and letters to the editor.

### 2.4. Data Extraction and Quality Assessment

Data extraction was systematically conducted by two distinct reviewers (M.P. and S.F.) utilizing a pre-defined data extraction form developed by L.S. The third reviewer (N.Z.-L.) was engaged to mediate any discrepancies or disputes between the reviewers. The data extracted from the eligible publications included various study characteristics, such as the first author’s name, publication year, country of origin, study design, and research groups. Additionally, specific information pertaining to pregnant women was collected, including the number of participants, their age, the gestational weeks at which PPROM occurred, and the microbiological profile observed among the different research groups. The Newcastle-Ottawa Quality Scale (NOS) was used to evaluate the methodological quality of the papers included in the analysis. The NOS assesses a study’s quality based on three criteria: selection, comparability, and exposure, with a maximum number of four, two, and three stars that may be granted to each criterion, respectively. Research studies that obtained scores of 7 or above on the NOS were deemed to be of a high level of quality [17].

## 3. Results

**Figure 1:**
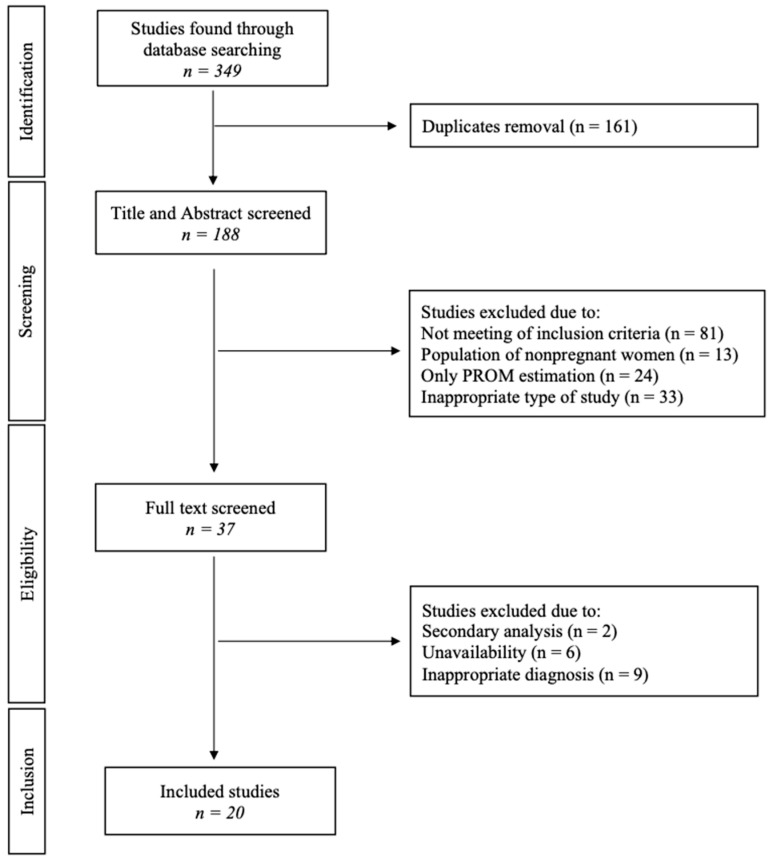
Flow diagram of the study selection.

The flow diagram in Figure 1 shows a concise overview of the comprehensive study selection procedure. Initially, a comprehensive database search yielded a total of 349 articles. Following the removal of duplicates, a total of 188 articles were subjected to the first screening based on their titles and abstracts. A total of 37 articles were chosen for a comprehensive review of their full-text content based on the relevance of their abstracts and titles.

This systematic review included a total of 20 studies [18,19,20,21,22,23,24,25,26,27,28,29,30,31,32,33,34,35,36,37]. Among the included papers, four studies were conducted by Kacerovsky et al. in the Czech Republic [23,24,27,37], three were performed in the UK [26,31,32], three papers were based on data from the Chinese population [18,20,33], two studies were conducted in Egypt [22,28], one in Switzerland [19], one in Italy [35], one in USA [36], one in Canada [34], one in India [29], one in Saudi Arabia [21], one in Vietnam [25], and one in Korea [30]. The investigations incorporated a collective sample size of 7137 pregnant women, of which 2157 experienced PPROM and 4980 had uncomplicated pregnancies resulting in term deliveries. The occurrence of PPROM ranged from 22 + 0 to 36 + 6 weeks of gestation. The microbiome was predominantly analyzed using 16S rRNA sequencing, targeting the V1–V2, V3–V4, or V3–V5 regions, as well as conventional microbiological analysis. Detailed characteristics of included studies can be found in Table 1. The overall risk of bias in the included studies were assessed to be minimal.

## 4. Discussion

The present study provides a thorough examination of the microbiota composition in pregnant women, revealing a significant correlation between an altered vaginal microflora and an increased risk of PROM [18,20,21,22,25,26,28,30,31,32,33,35]. There is a single study where no such correlation was identified; in this particular research, vaginal swabs were collected before and after fetal spina bifida repair surgery, which were conducted between 20 and 25 WG [19].

The most important change is the decrease in the natural vaginal populations of *Lactobacillus* spp. as well as *Weissella* spp., and *Rickettsia* spp., which are associated with term deliveries [30]. This reduction disturbs the natural balance of the vaginal environment, facilitating the proliferation of potentially harmful species. Vaginal dysbiosis has been associated with an increased risk of PPROM and subsequently PTB [20,26,32]. Additionally, the presence of certain bacteria, such as *Sneathia* spp., *Prevotella* spp., *Prevotella bivia*, *Prevotella timonensis*, *Peptoniphilus*, *Streptococcus* spp., *Dialister* spp., *Lactobacillus iners*, *Gardnerella vaginalis*, *Ochrobactrum* spp. and *Ureaplasma parvum* has been linked to an increased risk of PPROM [20,31,36]. Among these, *Ochrobactrum* spp. deserves particular attention due to its clinical relevance [38]. These non-enteric, Gram-negative bacilli are closely related to *Brucella* spp. and have been identified as opportunistic pathogens, especially in hospital environments [39]. Their association with infections in patients with indwelling medical devices and their resistance to penicillins and other antibiotics pose potential challenges in clinical management [38]. Although their role in PPROM is not yet fully understood, their presence in the vaginal microbiota may reflect underlying dysbiosis or increased susceptibility to secondary infections. Recent research has clarified the distinct functional roles of *Prevotella bivia* and *Prevotella timonensis* within the vaginal microbiome, in the context of BV, which is a known risk factor for PPROM [40]. *P. bivia* is frequently detected in women with BV and contributes to microbial imbalance [41]. Despite its abundance, it does not trigger a strong inflammatory response from vaginal epithelial cells, which aligns with its ability to evade immune detection and participate in biofilm formation [42]. This immune evasion may facilitate persistent colonization and microbial shifts associated with membrane weakening. In contrast, *P. timonensis* exhibits strong adhesion to vaginal and endocervical epithelial cells, comparable to *Gardnerella vaginalis*, another key BV-associated species [40]. However, it does not provoke a comparable proinflammatory reaction. Genomic analysis of *P. timonensis* has revealed a unique repertoire of mucus-degrading enzymes, including four putative fucosidases and two sialidases [40]. These enzymes compromise mucosal integrity and promote colonization by other microorganisms, including potential pathogens [40]. The presence and activity of *P. timonensis* contribute to epithelial vulnerability and microbial synergy, which are relevant in the pathogenesis of PPROM. Functional profiling of such species is essential for understanding their role in pregnancy complications. Other species considered detrimental that could lead to PPROM were *Megasphaera* spp., *Faecalibacterium* spp., *Bifidobacterium* spp., *Xanthomonadales* spp., *Gammaproteobacteria* spp., *Alphaproteobacteria* spp. [18], *Bacteroides* spp., *Sphingomonas* spp. [30], *Streptococcus agalactiae*, *Escherichia coli*, *Staphylococcus aureus* [22,35], *Chlamydia trachomatis*, *Ureaplasma urealyticum* [33] or Group B *Streptococcus* [21,33].

It is also known that antibiotic treatment and vaginal microbiome eradication significantly reduce the number of harmful bacteria and protect against PPROM [32,35,43]. A reduction in the population of *Lactobacillus* spp. and a subsequent rise in vaginal bacterial diversity preceded the rupture of fetal membranes and persisted after membrane rupture. Such change in vaginal microbiome could serve as an early indicator for the risk of PPROM [31,32,36]. The protective role of lactobacilli, with the exception of *L. iners*, against the incidence of PTB and PPROM was demonstrated [26]. Notably, *L. mulieris* specifically was linked to a reduced risk of PPROM [18].

According to a Cochrane review from 2017, the need for intervention in cases of PPROM or symptoms of vaginal infection is apparent [44]. A study by Ambalpady et al. has shown that antimicrobial treatment should be initiated based on the established changing microbiological pattern and be appropriate to the geographic region, and should be based on the results of the microbiological vaginal swab [43]. Prophylactic treatment for women with PPROM might include the use of ampicillin, erythromycin, or their combination, as well as the combination of erythromycin and azithromycin [45]. However, routine vaginal swab testing is not advised by the National Institute for Health and Care Excellence (NICE), American College of Obstetricians and Gynecologists (AJOG), Society of Obstetricians and Gynaecologists of Canada (SOGC) and Royal College of Obstetricians and Gynecologists (RCOG) [46,47,48,49]. While antibiotic therapy post-PPROM has been shown to correlate with positive outcomes, further investigation is needed into the prophylactic use of antibiotics in women at an increased risk of PPROM or PTB. Conversely, the study conducted by Genovese et al. indicates the administration of metronidazole and clotrimazole does not significantly lower the risk of PROM [35]. No other studies have assessed the effects of antibiotic treatment or prophylactic use in women at risk of PPORM. On the other hand, Malla et al. proposed the use of imipenem and amikacin as effective treatment options post-PPROM for Gram-negative bacteria and linezolide and vancomycin for Gram-positive bacteria. This approach was reported to be effective in treating amniotic infections [29]. However, there are no other reports of successful chorioamnionitis antibiotic treatment. Managing amniotic infections complicated with chorioamnionitis is challenging and carries the risk of preterm delivery, intrauterine fetal demise and could even lead to a severe maternal infection [45].

The use of erythromycin resulted in correcting vaginal dysbiosis in around 50% of the cases [32], while metronidazole and clotrimazole did not significantly reduce the incidence of PPROM [35]. However, the administration of antibiotics, such as erythromycin, may reduce the overall number of *Lactobacilli* spp. Antibiotic treatment did not yield a statistically significant effect on the relative abundance of *Prevotella*, *Lactobacillus*, or *Peptoniphilus* (following the administration of ampicillin or amoxicillin combined with azithromycin). However, a significant decrease in the abundance of *Weeksella*, *Lachnospira*, *Achromonacter*, and *Pediococcus* was seen after this course of antibiotics. Conversely, there was a substantial rise in the presence of *Peptostreptococcus* and *Tissierellacea* [36]. Since the dominant bacterial species remains uncertain after a specific antibiotic therapy [1], it seems reasonable to consider the use of probiotics during or following antibiotic therapy. The use of probiotic strains of *Lactobacillus fermentum* (CECT5716) [50], *L. rhamnosus* (DSM14870) and *L. gasseri* (DSM14869) [51] following PPROM has the potential to extend the duration of pregnancy. Nevertheless, no studies have been conducted to assess the effectiveness of probiotics in pregnant women at risk of PTB or PPROM. Further research could improve outcomes and decrease the rates of PTB and PPROM.

There have been papers discussing the potential application of bacterial microflora in pregnant women as a biomarker for PPROM and PTB. The findings by Mu et al. and Yan et al. contribute valuable insight into the efficacy of innovative biomarkers or screening methods for the early detection of PPROM [18,20]. The investigation conducted by Yan et al. demonstrated that *Lactobacillus crispatus*, *Lactobacillus iners*, *Lactobacillus gasseri*, *Gardnerella vaginal*, *Prevotella bivia*, *Ochrobactrum* spp., *Prevotella timonensis*, and *Ureaplasma parvum* could be predictive of PPROM [18,20]. Almaghrabi and Hussein have shown that Group B *Streptococcus* could also be used in PPROM prediction [21]. Furthermore, in the aforementioned studies, there was a correlation between BV, particularly aerobic bacteria, and the occurrence of PTB following PPROM [22,25,28,31,35,36]. Yet, Jayaprakash et al. did not find the association between vaginal microbiota and pregnancy latency [34].

The precise mechanisms by which the vaginal microbiota can affect PPROM and PTB remain not fully understood. However, potential pathways may involve modulatory effects on the immune system, inflammatory processes, and the synthesis of hormones that play a crucial role in the maintenance of pregnancy. As previously mentioned, one possible factor contributing to the incidence of PPROM is a shift in the composition of vaginal microbiota prior to the onset of PPROM. However, it has been noted that the bacterial microflora changes when exposed to the outflow of amniotic fluid. This can be attributed to the changes in vaginal pH as well as elevated levels of glucose and other nutrients that bacteria thrive on. The study demonstrated that *Prevotella*, *Peptoniphilus*, *Streptococcus*, and *Dialister* were found to be the predominant bacterial species in the swabs taken after PPROM [31]. Jayaprakash et al. have demonstrated that the abundance of *Weeksella*, *Lachnospira*, *Achromobacter*, and *Pediococcus* is dramatically diminished when *Peptostreptococcus* and *Tissierellaceae* become dominant after PPROM, regardless of antibiotic treatment [34]. While further research is needed to fully understand the relationship between the vaginal microbiome and PPROM, maintaining a balanced vaginal microbiome is vital for reducing the risk of PTB and other complications during pregnancy, as well as developing effective strategies for preventing PPROM.

Kacerovsky et al. conducted a total of four studies examining the relationship between microbiota and the risk of PPROM, as well as the infiltration of bacteria into the amniotic cavity and the subsequent effects on neonatal outcomes [23,24,27,37]. Previous studies have demonstrated that *Ureaplasma* and *Gardnerella vaginalis* are the predominant bacteria associated with intrauterine invasion. Conversely, *Lactobacillus crispatus* has been found to exert a protective effect against amniotic fluid infection [37]. The correlation between bacterial microbiota and neonatal sepsis was shown in previous research, with predominant bacteriological findings within the cohort consisting of *Staphylococci*, *Ureaplasma*, *Candida albicans*, and *Streptococcus viridans* [52]. While *Lactobacillus* spp. is generally considered protective in the vaginal microbiome, recent findings suggest that their role may be more nuanced than previously thought. For instance, *Lactobacillus crispatus* has been associated with vaginal health and resistance to pathogenic colonization [53]. However, Kacerovsky et al. reported a higher prevalence of *L. crispatus* in PPROM cases, which appears contradictory to the established notion that reduced *Lactobacillus* abundance correlates with increased risk [37]. This discrepancy may be explained by several factors, including differences in sampling time (e.g., before vs. after membrane rupture), host immune status, or co-occurrence with other microbial species [18]. It is plausible that *L. crispatus* may be present in early stages of dysbiosis or as a compensatory response to microbial imbalance, and its protective effect may be context dependent [54]. These findings underscore the importance of strain-level and functional analyses rather than relying solely on genus-level associations. While antibiotic therapy remains a cornerstone of PPROM management, its impact on beneficial microbial populations such as *Lactobacillus* spp. must be carefully considered [55]. The use of targeted probiotics, particularly strains like *L. fermentum*, *L. rhamnosus*, and *L. gasseri*, may offer a complementary strategy to restore microbial balance and potentially prolong pregnancy duration [56]. However, randomized controlled trials are needed to validate their efficacy and safety in pregnant populations.

This study is the first comprehensive evaluation of the impact of microbiota on PPROM rates. However, it is essential to acknowledge that this review does have several limitations. The number of published articles examining the association between vaginal microbiota and the occurrence of PPROM is limited and characterized by small study cohorts. As initially intended by the inclusion criteria, it is possible that included studies did not directly compare PPROM cases to control groups. However, these investigations yielded useful information that made it possible to synthesize the findings and make indirect comparisons. Utilizing high-quality observational data is still an acceptable strategy to provide significant insights and steer future research directions, despite the difficulty and ethical limitations of performing experiments with matched controls in this situation. Furthermore, the publications that were included in the analysis predominantly consisted of retrospective studies, with only short pregnancy periods used in prospective studies to assess the subsequent swab outcomes. This underscores the need for further follow-up investigations for more conclusive outcomes. Another limitation stems from the diversity of the evaluated outcomes. Various bacterial strains were analyzed, and the samples were not collected during the same stage of pregnancy. Furthermore, existing scientific literature suggests that the composition of vaginal microbiota changes after PPROM [31]. Hence, analyzing samples obtained after the outflow of amniotic fluid may introduce bias when trying to establish a causal relationship between the observed results since it is difficult to ascertain if these changes are the precursor or a consequence of PPROM. It is essential to acknowledge that the timing of sample collection might have a substantial impact on the findings. Despite these limitations, the observed patterns suggest a likely association between the presence of specific bacteria present in the vaginal microflora and the incidence of PPROM, as well as the duration of pregnancy [22,25,28,31,35,36]. However, in spite of these encouraging results, additional research is required to examine the extent of the relationship between the bacterial microbiome and the risk of PPROM, as well as the duration of pregnancy. The microbial composition undergoes significant shifts following the rupture of membranes, influenced by changes in vaginal pH, nutrient availability, and exposure to amniotic fluid [57]. This temporal variability complicates the interpretation of microbial data and calls for longitudinal studies that capture microbial transitions before, during, and after PPROM [58]. The novelty of our study lies in its integrative approach, synthesizing data from diverse sources to identify consistent microbial patterns associated with PPROM. Unlike previous reviews, we emphasize the potential of microbiome-informed predictive models, including the use of artificial intelligence to stratify risk and guide clinical decision-making.

None of the included studies used artificial intelligence to develop the predictive model for PPROM or PTB. Previous research has demonstrated a limited availability of biomarkers suitable for integration into the artificial intelligence model development [59]. Nevertheless, Zhang et al. demonstrated using the Bayesian stepwise discriminant analysis model that the integration of bacterial microflora and clinical data has the potential to predict PPROM with an accuracy rate ranging from 85% to 87%. This study examines the significant impact of *U. urealyticum*, *C. trachomatis*, and Group B *streptococci* on PPROM rates [33]. The implementation of artificial intelligence algorithms to analyze risk-based microbiological patterns in patients with PPROM has the potential to improve perinatological outcomes. In the context of high-risk pregnancies, performing routine microbiological assessments and implementing appropriate therapeutic measures may potentially lower PPROM rates. Furthermore, probiotics administered vaginally could also contribute to lowering the risk of PPROM, and additionally extend the duration of pregnancy or prevent the invasion of pathogens into the amniotic cavity after PPROM.

## 5. Conclusions

This study provides a comprehensive analysis of the microbial composition in pregnant women, demonstrating a correlation between disturbances in the bacterial microflora and an increased risk of PPROM. One significant change includes the reduction in levels of naturally occurring vaginal strains of *Lactobacillus* spp., *Weissella* spp., and *Rickettsiales* spp. This was accompanied by the dominance of other bacterial species such as *Sneathia* spp., *Prevotella* spp., *Prevotella bivia*, *Prevotella timonensis*, *Peptoniphilus*, *Streptococcus* spp., *Dialister* spp., *Lactobacillus iners*, *Gardnerella vaginalis*, *Ochrobactrum* spp., *Megasphaera* spp., *Faecalibacterium* spp., *Bifidobacterium* spp., *Xanthomonadales* spp., *Gammaproteobacteria* spp., *Alphaproteobacteria* spp., *Bacteroides* spp., *Sphingomonas* spp., *Streptococcus agalactiae*, *Escherichia coli*, *Staphylococcus aureus*, *Chlamydia trachomatis*, *Ureaplasma urealyticum*, *Ureaplasma parvum or* Group B *Streptococcus* leading to PPROM. Recognizing the microbial patterns could lead to the development of risk-based microbiological interventions and probiotic treatment, potentially improving the management and outcomes of patients with PPROM.

## Figures and Tables

**Table 1:** Vaginal microbiome as a potential biomarker in PPROM.

Study	Population and Tested Microbes	Study Design	Sample Collecting	Outcome	NOS
Mu et al., 2023 [18]	310 pregnant women in the early II. trimester (6 PPROM cases, 46 PROM and 255 healthy pregnancies) *Lactobacillus* spp. *Megasphaera* spp. *Faecalibacterium* spp. *Bifidobacterium* spp. *Xanthomonadales* spp. *Gammaproteobacteria* spp. *Alphaproteobacteria* spp.	Case-cohort study from China	Vaginal swabs analyzed with V3–V4 region of 16S rRNA gene on an Ion S5TM XL instrument.	Reduced risk of PPROM associated with *Lactobacillus mulieris* (adjusted odds ratio [aOR] 0.35, 95% confidence interval [CI]: 0.17–0.72). PPROM risk associated with *Megasphaera* spp. (aOR 2.27, 95%CI: 1.09–4.70), *Faecalibacterium* spp. (aOR 3.29, 95%CI: 1.52–7.13), *Bifidobacterium* spp. (aOR 3.26, 95%CI: 1.47–7.24), *Xanthomonadales* spp. (aOR 2.76, 95%CI: 1.27–6.01), *Gammaproteobacteria* spp. (aOR 2.36, 95%CI: 1.09–5.14) and *Alphaproteobacteria* spp. (aOR 2.45, 95%CI: 1.14–5.26).	7
Tevaearai et al., 2022 [19]	99 women undergoing fetal spina bifida repair between 20 + 6 and 25 + 5 WG. (48 PPROM cases) *Lactobacillus* Desquamative inflammatory vaginitis: *Gardnerella vaginalis*, *Escherichia coli* group B *Streptococcus* *Staphylococcus aureus* *Enterococcus faecalis*	Prospective observational study from Switzerland	Vaginal swabs analyzed with wet mount microscopic test after antibiotic therapy.	Pre- (OR 1.57, 95%CI: 0.74–3.32) and post-surgical (OR 1.26, 95%CI: 0.62–2.55) abnormal vaginal flora is not associated with PPROM.	8
Yan et al., 2022 [20]	102 pregnant women (48 with PPROM between 24 + 0 and 36 + 6 WG and 54 healthy women delivered at term). *Lactobacillus iners* *Gardnerella vaginalis* *Prevotella bivia*, *Ochrobactrum* spp. *Prevotella timonensis* *Ureaplasma parvum*	Cross-sectional study from China	Vaginal swabs analyzed with V3–V4 region of t16S rRNA genes Illumina NovaSeq PE250 platform and with conventional microbiological analysis.	*Lactobacillus crispatus*, *Lactobacillus iners*, *Lactobacillus gasseri*, *Gardnerella vaginal*, *Prevotella bivia*, *Ochrobactrum* spp., *Prevotella timonensis*, and *Ureaplasma parvum* play role in PPROM prediction (AUC 0.913, 95%CI: 0.86–0.97). *Ochrobactrum* spp. (AUC 0.89, 95%CI: 0.81–0.96), *Prevotella timonensis* (AUC 0.76, 95%CI: 0.67–0.86), *Gardnerella vaginal* (AUC 0.75, 95%CI: 0.65–0.84).	8
Almaghrabi and Hussein 2022 [21]	1201 pregnant women (969 with PROM and 232 with PPROM delivered at ≥27 WG). Group B *streptococcus* (72.9%) *Candida* spp. (18.6%).	Retrospective observational study from Saudi Arabia	Vaginal and rectal swabs analyzed with latex agglutination test, CAMP test, or automated identification machine	Group B *Streptococcus* dominates in Saudi women with membranes rupture (PPROM and PROM).	5
Elshabrawy et al., 2022 [22]	640 pregnant women (320 PPROM cases and 320 healthy pregnant women) *S. agalactiae* (25.5% PPROM) *E. coli* (25.0% PPROM) *Staphylococcus aureus* (16.9% PPROM)	Case control study from Egypt	Vaginal swabs analyzed with latex agglutination test and Yeast growth test.	Bacterial vaginosis (OR 6.3, 95%CI: 4.2–9.6), aerobic vaginitis (OR 39.7, 95%CI: 14.4–109.3), and vaginal candidiasis (OR 13.5, 95%CI: 3.2–57.4) relate to PPROM occurrence.	7
Kacerovsky et al., 2022 [23]	217 pregnant women with PPROM between 24 + 0 and 33 + 6 WG. *Ureaplasma* spp. *Mycoplasma hominis* *Chlamydia trachomatis*	Retrospective observational study from Czech Republic	Cervical fluid swabs analyzed with AmpliSens^®^ to detect bacterial DNA.	*Ureaplasma* spp. was present in 61% of PPROM cases and correlated with the presence of intra-amniotic infection and colonization.	7
Kacerovsky et al., 2021 [24]	405 pregnant women with PPROM between 24 + 0 and 33 + 6 WG. *Gardnerella vaginalis* (94%)	Prospective observational study from Czech Republic	Vaginal swabs analyzed with QIAamp DNA Mini Kit to detect *G. vaginalis* DNA and 16S rRNA region amplification technique.	*G. vaginalis* infection is associated with microbial invasion of the amniotic cavity.	8
Nguyen et al., 2021 [25]	79 pregnant women delivered preterm (34 with PPROM between 24 + 0–33 + 6 WG and 45 cases with preterm labor and intact membranes). *Lactobacillus* spp. *Mobiluncuss* spp. *Candida* spp. *Gardnerella vaginalis* *Trichomonas vaginalis*	Case control study from Vietnam	Vaginal swabs analyzed with Gram stain (bacteria) and wet mount (fungus).	Bacterial vaginosis was associated with preterm labor (OR 3.2, 95%CI: 1.2–8.2). Isolated aerobic bacteria were associated with premature rupture of membranes (OR 5.5, 95%CI = 2.1–14.1).	6
Goodfellow et al., 2021 [26]	254 pregnant women at 15 + 1–18 + 6 WG and 19 + 0–23 + 0 WG (109 with history of PPROM and 145 with low risk of PPROM). (22 women with PPROM). *Lactobacillus* spp.: *Lactobacillus iners* (33%) *Lactobacillus crispatus* (21.1%) Other (20.2%)	Case-control study from UK	Vaginal swabs analyzed with V3–V4 region of 16S rRNA gene on HiSeq 2500 Illumina platform	*L. iners*-domination is related to PPROM occurrence in II. trimester (aOR 3.44, 95%CI 1.06–11.15). sPTB/PPROM. Domination of *Lactobacilli*, but not *L. iners* may protect pregnant women from disbacteriosis and developing PPROM.	5
Kacerovsky et al., 2020 [27]	311 pregnant women with PPROM between 24 + 0 and 33 + 6 WG. *Lactobacillus crispatus* *Lactobacillus iners*.	Retrospective observational study from Czech Republic	Cervical swabs analyzed with 16S rRNA region amplification technique.	*L. crispatus*-domination in PPROM patients is related to decreased risk of intra-amniotic bacterial invasion.	6
Hassan et al., 2020 [28]	600 pregnant women (100 with aerobic vaginitis and 500 with normal flora), (28 PPROM cases, 51 PROM cases, 52 PTB cases).	Prospective observational study from Egypt	Vaginal swabs analyzed with Gram stain assessment.	Correlation between aerobic vaginitis and with: PTB (aOR 3.06, 95% CI 1.58–5.95), PROM (aOR 6.17, 95% CI 3.24–11.7), and PPROM (aOR 1.73, 95%CI: 0.68–4.4).	6
Malla et al., 2020 [29]	60 pregnant women with PPROM. *Enterococcus faecalis* (38.8%) *Escherichia coli* (27.7%) *Staphylococcus aureus* (11.1%) *Klebsiella pneumoniae* (12.9%) *Pseudomonas* *ạeruginosa* (3.7%) *Proteus mirabilis* (5.6%)	Cross-sectional study from India	Vaginal swabs analyzed with unknown test.	Imipenem (88.8%) and Amikacin (66.6%) are effective against Gram-negative bacteria. Linezolid (70.3%) and Vancomycin (55.5%) are effective against Gram-positive bacteria.	2
You Y.-A. et al., 2019 [30]	58 pregnant women (41 with PTB, 17 with PPROM 22 + 0 and 36 + 6 WG and 14 term deliveries). *Lactobacillus* spp. *Bacteroides* spp. *Sphingomonas* spp. *Weissella* spp. *Rickettsiales* spp.	Prospective observational study from Korea	Vaginal swabs analyzed with V3–V4 region of 16S rRNA gene on MiSeq Illumina platform.	*Bacteroides* spp. (22.8%) and *Sphingomonas* spp. (3.9%) are associated with PPROM (*p* < 0.01). *Weissella* spp. and *Rickettsiales* spp. associated with term deliveries.	6
Brown et al., 2019 [31]	1505 pregnant women between 6 + 0 and 10 + 0 WG. 502 high PTB risk (38 PPROM cases) and 1003 low PTB risk (22 PPROM cases) Total 60 PPROM cases. *Lactobacillus* spp. *Prevotella* spp. *Peptoniphilus* spp. *Streptococcus* spp. *Dialister* spp.	Prospective observational study from UK	Cervico-vaginal swabs from the posterior fornix taken at 12–17+6, 18–23+6, 24–29+6, 30–36+6 weeks+days analyzed with V1–V2 regions of 16S rRNA gene on MiSeq Illumina platform.	*Lactobacillus* spp. reduction and high vaginal bacterial diversity as an early risk factor for PPROM. High vaginal diversity and reduced *Lactobacillus* spp. abundance observed prior to PPROM in 20% and 26% of women at low and high risk of PTB respectively, and in only 3% of women delivered terminally. Higher vaginal diversity and instability of bacterial community during the second trimester associated with PPROM. *Prevotella*, *Peptoniphilus*, *Streptococcus*, and *Dialister* increased in PPROM vaginal microbiome.	8
Brown et al., 2018 [32]	First cohort (2013–2014): 250 pregnant women between 8 + 0 and 12 + 0 WG (15 PPROM cases). Second cohort (2013–2015): 87 women with PPROM. *Lactobacillus* species: *L. iners* (>92%) *L. crispatus* (>93%) *L. gasseri* (>80%) *L. jensenii* (>92%) *L. iners* (33–68%) *L. crispatus* (51–78%)	Prospective observational study from UK	Cervico-vaginal swabs from the posterior fornix taken at 8–12, 19–25, 27–30 and 32–36 WG analyzed with V1–V2 regions of 16S rRNA gene on MiSeq Illumina platform.	Vaginal microbiota composition is a risk factor for PPROM. *Lactobacillus* spp. reduction and vaginal dysbiosis is observed prior to the PPROM and persisted following membrane rupture. *Lactobacillus* reduction and increased abundance of *Sneathia* spp. associated with fungal infections and neonatal sepsis. Erythromycin treatment eradicated vaginal dysbiosis in 47% of cases.	8
Zhang et al., 2017 [33]	220 pregnant women (112 with PPROM and 108 healthy pregnant women between 28 + 0 and 36 + 6 WG). *Chlamydia trachomatis* *Ureaplasma urealyticum* *Candida albicans* group B *streptococci* *herpes simplex virus*-1 (*HSV*-1) and *HSV*-2	Case control study from China	Vaginal swabs analyzed with QIAamp MiniStool kit and RT-PCR.	Analysis of included bacteria could predict PPROM with 84.1–86.8% accuracy. *U. urealyticum* (11.6% vs. 3.7%), *C. trachomatis* (17.0% vs. 5.6%), and group B *streptococci* (22.3% vs. 6.5%) has most meaningful impact on PPROM prediction.	7
Jayaprakash et al., 2016 [34]	51 pregnant women with PPROM between 24 + 0 and 33 + 6 WG. *Mycoplasma* spp. *Ureaplasma parvum* *U. urealyticum* Group B *Streptococcus* (5.9%) *Lactobacillus crispatus* *L. iners* *Prevotella timonensis* *Gardnerella vaginalis* *Corynebacterium* spp. *Escherichia coli*	Prospective cohort study from Canada	Vaginal swabs analyzed with pyrosequencing of the *cpn* 60 on the 454 GS FLX Titanium and GS Junior sequencing platforms.	Vaginal microbiota in patients with PPROM did not correlate with pregnancy latency duration. *Megasphaera* type 1 and *Prevotella* spp. detected in all vaginal samples. *Lactobacillus* domination was in 18.6% of samples.	8
Genovese et al., 2016 [35]	600 pregnant women between 28 + 0 and 32 + 0 WG.: - Women with dysbacteriosis (55%) - women with lactobacillus domination (45%) - 8 women with PPROM. *Lactobacillus* spp. (17 morphotypes) *Candida albicans* (27.7%) *Enterococcus* spp. (28.6%) *Escherichia coli* (25.5%) *Gardnerella vaginalis* (22.8%) *Peptococcus* spp. (21.8%) *Candida non albicans* (11.7%)	Retrospective observational study from Italy	Vaginal swabs analyzed to evaluate the Lactobacillary grade on Schroder’s classification.	Bacterial vaginitis (*E. coli*, *Enterococcus* spp., *Peptococcus* spp., *G. vaginalis*) is related with PPROM occurrence. Bacterial eradication with metronidazole and clotrimazole was insignificant in PPROM risk reduction (RR 0.51, 95%CI: 0.12–2.11).	6
Baldwin et al., 2015 [36]	27 pregnant women at 23 + 1–34 + 5 WG (15 with PPROM and 12 healthy pregnant women).	Retrospective observational study from USA	Vaginal swabs analyzed with V3–V5 region of 16S rRNA on MiSeq 600 Illumina	*Lactobacillus* spp. decrease in PPROM women. *Prevotella* and *Peptoniphilus* became most dominant in cases without antibiotic treatment. *Weeksella*, *Lachnospira*, *Achromobacter*, and *Pediococcus* significantly reduced, *Peptostreptococcus* and *Tissierella* dominate during and after the antibiotic treatment.	7
Kacerovsky et al., 2015 [37]	61 pregnant women with PPROM between 24 + 0 and 36 + 6 WG. *Lactobacillus* dominated: *Lactobacillus crispatus* (*n* = 25) *Lactobacillus gasseri* (*n* = 13) *Lactobacillus iners* *Lactobacillus jensenii* non-*Lactobacillus* dominated: *Ureaplasma* spp., *Propionibacterium acnes*, *Fusobacterium nucleatum*, *Veillonela* spp., *Streptococcus* spp., *Haemophilus influenzae* (*n* = 11) *Gardnerella vaginalis* and *Sneathia sanguinegens* dominated (*n* = 12)	Prospective observational study from Czech Republic	Cervical and amniotic fluid swabs analyzed with 16S rRNA gene sequencing on GS FLX + sequencer.	Non-*Lactobacillus* CSTs associated with a strong cervical inflammatory response and higher rates of microbial invasion of the amniotic cavity. *L. crispatus* occurs more often in PPROM cases and is connected to a low rate of microbial invasion of the amniotic cavity.	8

prelabor rupture of membranes—PROM; preterm premature rupture of membranes—PPROM; amniotic cavity—MIAC; community state types—CSTs; term delivery—TD; preterm birth—PTB; lactobacillary grade—LBG; fetal spina bifida—fSB; bacterial vaginosis—BV; healthy vaginal flora—HVF; inflammatory vaginitis—DIV.

## Data Availability

The data that support the findings of this study are available on request from the corresponding author (S.F.).

## Supplementary Materials

The following supporting information can be downloaded at: https://www.mdpi.com/article/10.3390/jcm14186577/s1, Supplementary Table S1: PRISMA checklist
